# Ischemic Heart Disease and Rheumatoid Arthritis—Two Conditions, the Same Background

**DOI:** 10.3390/life11101042

**Published:** 2021-10-03

**Authors:** Elena Rezuș, Luana Andreea Macovei, Alexandra Maria Burlui, Anca Cardoneanu, Ciprian Rezuș

**Affiliations:** 1 Department of Rheumatology and Rehabilitation, Grigore T. Popa University of Medicine and Pharmacy, 700115 Iași, Romania; elena.rezus@umfiasi.ro (E.R.); maria-alexandra.burlui@umfiasi.ro (A.M.B.); anca.cardoneanu@umfiasi.ro (A.C.); 2 Department of Internal Medicine, Grigore T. Popa University of Medicine and Pharmacy, 700115 Iași, Romania; ciprian.rezus@umfiasi.ro

**Keywords:** rheumatoid arthritis, atherosclerosis, inflammation, cardiovascular disease, traditional risk factors, cardiovascular risk assessment, dyslipidemia

## Abstract

Rheumatoid arthritis (RA) is one of the most frequent inflammatory rheumatic diseases, having a considerably increased prevalence of mortality and morbidity due to cardiovascular disease (CVD). RA patients have an augmented risk for ischemic and non-ischemic heart disease. Increased cardiovascular (CV) risk is related to disease activity and chronic inflammation. Traditional risk factors and RA-related characteristics participate in vascular involvement, inducing subclinical changes in coronary microcirculation. RA is considered an independent risk factor for coronary artery disease (CAD). Endothelial dysfunction is a precocious marker of atherosclerosis (ATS). Pro-inflammatory cytokines (such as TNFα, IL-1, and IL-6) play an important role in synovial inflammation and ATS progression. Therefore, targeting inflammation is essential to controlling RA and preventing CVD. Present guidelines emphasize the importance of disease control, but studies show that RA- treatment has a different influence on CV risk. Based on the excessive risk for CV events in RA, permanent evaluation of CVD in these patients is critical. CVD risk calculators, designed for the general population, do not use RA-related predictive determinants; also, new scores that take into account RA-derived factors have restricted validity, with none of them encompassing imaging modalities or specific biomarkers involved in RA activity.

## 1. Introduction

Rheumatoid arthritis (RA) refers to the most common inflammatory rheumatic disease characterized by synovitis of small- and medium-sized joints, bone erosions, and ultimately destruction and loss of joints function. Aside from affecting the joints, RA may evolve with systemic involvement (respiratory, hematologic, cardiovascular), often in the case of a long-term-evolution disease or inadequate disease control [1].

RA affects 0.5–1.0% of the global population. Women are more frequently affected than men (women/men ratio 3/1); this disease occurs more commonly in the fourth or fifth decades of life [1]. 

Patients with RA present an increased risk for ischemic and non-ischemic heart disease due to subclinical pathological alterations in heart muscle and coronary arteries. It is known that systemic inflammation is a significant contributing factor for the increased cardiovascular (CV) risk in patients with this disease [2,3] and is connected with arterial stiffness, lipid-salvage processes, and destabilization of atherosclerotic plaques [4,5].

RA is a chronic inflammatory disorder confirmed as being an independent CV risk factor by the European Society of Cardiology (ESC) guidelines [6]; therefore, the European League Against Rheumatism (EULAR) recommends a multiplication by 1.5 of cardiovascular disease (CVD) risk scores in individuals with RA. Additionally, ongoing EULAR guidelines indicate that rheumatologists are responsible for the evaluation and management of CVD risk in patients with RA [7].

Studies indicate that the prevalence of heart diseases in patients with RA is higher than in the general population and is equivalent to that in patients with type 2 diabetes [8,9]. An incidence of 30–60% of CVD is reported in RA patients, including heart failure (HF), ischemic disease, pericarditis, myocarditis, and cardiomyopathy [10]. The risk for myocardial infarction (MI) is 68% and for stroke is 41%, both these values being higher compared to the general population [11].

A study accomplished by A TransAtlantic Cardiovascular Consortium for Rheumatoid Arthritis (ATACC-RA) discovered that approximately 49% of CV incidents in RA were caused by traditional CVD risk factors (especially smoking and hypertension) and 30% by RA-related aspects, such as elevated DAS (Disease Activity Score) 28, positive rheumatoid factor (RF) and/or anti-citrullinated protein antibody (ACPA), and raised erythrocyte sedimentation rate (ESR) and C-reactive protein (CRP) [12].

In addition, CV events explain 42% of total RA-related deaths (representing the leading cause of mortality in RA) [13] and appear early in patients having RA; for that reason, their survival rate is reduced by 5–15 years [14], representing the major cause of mortality in RA [8,9].

## 2. Cardiac Involvement in Rheumatoid Arthritis

**Figure 1:**
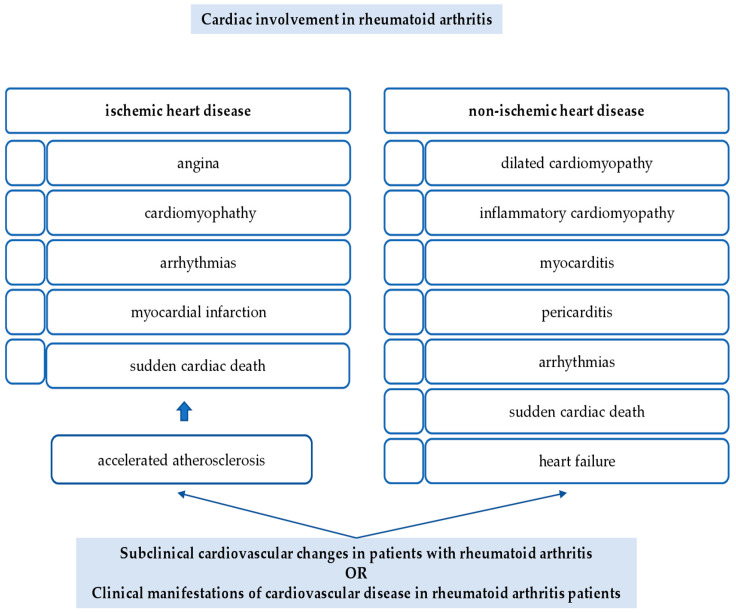
Cardiac involvement in rheumatoid arthritis. There are two main aspects of cardiovascular (CV) involvement in rheumatoid arthritis (RA): ischemic heart disease (IHD) and non-IHD. Clinical expression of ischemic heart disease is directly connected with the degree of ischemia, angina, cardiomyopathy, or arrhythmias in a less acute type and myocardial infarction (MI) and sudden cardiac death (SCD) in a more acute one. The most prevalent type of non-IHD is cardiomyopathy, dilated or inflammatory. Myocarditis may be diffuse necrotizing or granulomatous. Another pathological process is pericarditis, responsible for an important accumulation of fluid that may transform into a life-threatening state, cardiac tamponade, with an acute loss of ventricular function as a result of cardiogenic shock. A separate cardiac involvement is arrhythmia. It is well-known that individuals with RA have an increased CV morbidity and mortality rate, for example, the risk for SCD. Patients with RA have an increased risk for HF.

There are two main aspects of CV involvement in RA: ischemic heart disease (IHD) and non-ischemic heart disease (non-IHD), as shown in Figure 1. 

### 2.1. Ischemic Heart Disease

IHD, known as coronary heart disease (CHD), is a condition that produces an insufficient blood supply to the cardiac muscle. It is also named coronary artery disease (CAD) due to the role of coronary arteries in the process [5].

IHD is an important cause of CV death in patients with RA. It is demonstrated that the risk of MI in those patients is comparable to that in diabetes mellitus [15]. Studies indicate that the incidence of MI is 70%, being higher than in the general population. Moreover, patients with RA have poorer long-term outcomes compared with individuals without RA and a higher risk of death. Additionally, persons with RA have a two-fold increased risk of sudden cardiac death (SCD) [5].

Systemic inflammation, the main cause of CAD, is related to accelerate atherosclerosis (ATS) progression, with vascular endothelial dysfunction and abnormal lipid accumulation [16]. In RA, chronic inflammation is a key factor for dyslipidemia, with low levels of high-density lipoprotein cholesterol (HDL-C) and high levels of low-density lipoprotein cholesterol (LDL-C). In addition, RA and ATS are auto-inflammatory diseases, sharing numerous inflammatory cytokines, environmental factors, and genetic susceptibility [17].

Anatomically, in the process of ATS, atherosclerotic plaques reduce the lumen of arteries, decreasing blood flow. When those plaques are unstable and cause cleavage or a rupture, blood clots may form, obstructing coronary blood vessels and leading to acute coronary syndrome (ACS). A deficiency in oxygen supply in the myocardium generates a disturbance or a death of cardiomyocytes, the contractile cells of the cardiac muscle.

Clinical expression of CHD is directly connected with the degree of ischemia, with angina, cardiomyopathy, or arrhythmias in a less acute type and MI and SCD in a more acute one [5].

### 2.2. Non-Ischemic Heart Disease

Non-IHDs concerning CV problems appearing in the absence of CAD. In this category, inflammation in the myocardium or pericardium, systolic or diastolic dysfunction, myocardial fibrosis, conduction errors, and valvular anomalies are included, resulting in dilated or inflammatory cardiomyopathy. Usually, non-IHDs evolve gradually, being linked with variation in cellular composition and structure of the cardiac muscle [5].

The most prevalent type of non-IHD is cardiomyopathy, a condition with enlarged and rigid ventricles. Dilated cardiomyopathy, a progressive disease with different trigger factors (intra- or extra-cardiac), may eventually necessitate heart transplantation because of the degree of cardiac dysfunction. Often, biventricular dilatation is present, with systolic impairment and other valvular problems, embolus, and arrhythmias causing heart failure. Chronic inflammatory processes taking place in the myocardium (known as myocarditis) are the leading source of the disease. 

Myocarditis may be diffuse necrotizing or granulomatous. According to [18], the percentage of cardiomyopathy in patients with RA is believed to be 3–30%, discovered by autopsy. The precise etiopathogenesis of this disease is not fully understood, but some inflammatory cytokines (for example, interleukin (IL)-1α) and mediators are released from the degenerating myocardium, thus activating inflammatory corpuscles in other nearby cells [10]. Additionally, the toxicity of antirheumatic drugs may trigger a myocardial disturbance [19].

In RA, another pathological process is pericarditis, the inflammation of the pericardium, responsible for an important accumulation of fluid that may transform into a life-threatening state, cardiac tamponade, with an acute loss of ventricular function as a result of cardiogenic shock [5]. Pericarditis may be a complication of early RA or may appear before the rheumatological disease. 

A separate cardiac involvement in RA patients is arrhythmia, revealing conduction problems determined by local ischemia, rheumatoid nodules, amyloidosis, or HF.

It is well-known that individuals with RA have an increased CV morbidity and mortality rate, for example, the risk of SCD [20]. Inflammation is an independent risk factor for SCD [21], being at the origin of ventricular repolarization anomalies (for example, corrected QT interval prolongation, QT dispersion, and autonomic disturbance). Additionally, a higher sympathetic activity (present in patients with RA) is correlated to an abnormal heart rate [22]. Studies revealed that inflammation and CRP levels are linked to autonomic dysfunction, as well as QTc prolongation [23].

RA patients have an increased risk for HF [24]. It is presumed that the incidence of this complication is two-fold higher in patients with RA compared to the general population and prevails in women [25]. Davis et al. mentioned that the presentation and evolution of HF are not similar in patients with RA, compared with those without RA or other rheumatic inflammatory diseases. There are fewer distinctive signs and symptoms of HF in patients with RA, and ejection fraction is often preserved; they do not have a good response to treatment and generally have a poor prognosis, with a mortality risk two-fold higher for those with RA compared with non-RA patients [26].

In the evolution of RA, patients display first diastolic cardiac dysfunction [27]; in time, left ventricular diastolic disturbance develops, being associated with specific changes in electrocardiography (ECG). Diastolic impairment is strongly related to chronic systemic inflammation in RA, in particular with increased levels of CRP and ESR and with positive RF and ACPA [28].

In RA, the incidence of non- IHF is at least as common as the incidence IHF [5].

Cardiac involvement in patients with RA can extend from subclinical and asymptomatic changes to clinical manifestations of HF.

*Subclinical changes in RA patients’ hearts.* Most RA patients will not have important cardiac manifestations for many years. However, the CV system may often exhibit subclinical and asymptomatic changes, which particularly concern the myocardium and coronary system. A total of 50% of RA patients without a history of CVD have signs of cardiac fibrosis or inflammation on imaging or decreased left ventricle function [29]. Additionally, approximately 30% of those patients present with cardiac microvascular disorder [30].

Different non-invasive imaging instruments offer important information on the structure and function of the CV system [31]. Cardiac MRI (magnetic resonance imaging) and positron emission tomography-computed tomography (PET-CT) performed in patients with RA with no previous diagnosis of CVD revealed that up to half of them had evidence of cardiac inflammation or fibrosis [32]. A total of 50% of patients with RA without obvious signs of cardiac disease showed impaired systolic and diastolic left ventricle functions, demonstrating that the contractile function is frequently impaired.

Asymptomatic pericarditis and cardiac valvular disease are discovered in different echocardiographic investigations in RA patients [33]. 

One-third of RA patients without clinical CVD also have subclinical changes in coronary microcirculation, as measurements of myocardial flow reserve reveal [34]. Different types of coronary plaques are present even in the absence of CAD in RA patients. CT angiography is the main investigation confirming that hypothesis. 

Another aspect in those patients is represented by an increased risk of developing episodes of silent myocardial infarction [5].

In conclusion, it is mandatory to discover, prevent, and treat all these subclinical alterations of the myocardium and coronary system in patients with RA because of the complications that follow, ischemic and non-ischemic, which may be severe or even fatal [5].

*Clinical manifestations of heart failures in RA.* Studies confirm that especially new-onset RA are at increased risk of developing IHD and non-IHD [34]. Wang et al. published a systematic review and meta-analysis in 2020 comparing CV presentation and evolution in patients with RA and CVD. Data of this study demonstrated that there is a 40–50% increased risk for mortality because of CVD in RA patients, including congestive HF (CHF) and SCD [35,36]. Two studies published by Myasoedova et al. and Arts et al. reached the conclusion that RA disease activity significantly affects CV risk; thus, each period of intensified activity is related with a 7% increased CV risk, and a decreased activity status lowers the risk [37,38].

Different proinflammatory cytokines are implicated in the etiopathogenesis of heart implication in RA: tumor necrosis factor (TNF)-α, IL-1β, IL-6, and IL-17 [5]. Specific mechanisms implicated in non-IHD disease are still not fully known compared to those concerning IHD [29].

## 3. Cardiovascular Risk Factors in the Rheumatoid Arthritis Population

### 3.1. Traditional Risk Factors

In patients with RA, both traditional CV risk factors and RA-related characteristics, such as chronic inflammation and side effects of medications, play an important role in CVD-related morbidity and mortality [7,39,40]. Studies confirm that the prevalence of traditional risk factors in the RA population is higher compared with the general population, with hypertension, hyperlipidemia, diabetes, and smoking representing the most important ones, but the risk profile, in this case, is a lot different; some researchers are even talking about a “risk factor paradox” in patients with RA [39,40].

Boyer et al., in 2011 [41], published a meta-analysis of 15 case–control studies with 2956 RA patients and 3713 controls, which demonstrated that the RA group presented a higher prevalence of traditional CV factors, particularly smoking and low levels of HDL-C. Additionally, Chung et al. suggested that the prevalence of metabolic syndrome is higher in patients with RA, this fact being directly related to a two-fold increase in the risk of CVD [42].

#### 3.1.1. Smoking 

Smoking is known to be an important common risk factor for CVD in RA [43,44]. In a study by Rojas-Serrano et al., patients with a history of smoking presented as RF- and ACPA-positive and had more severe progress of RA [45]. In addition, Baghdadi et al. conducted a meta-analysis that demonstrated an increased CV risk in such patients [39]. Smoking influences the expression of inflammatory genes implicated in RA pathogenesis [45] and is responsible for gene regulation and gene methylation involved in the development of CAD [46].

#### 3.1.2. Hypertension

It is demonstrated that, in RA patients, high blood pressure outlines an independent indicator of CVD. A COMORA study indicated that arterial hypertension in the RA population exists in approximately 40% [47,48]; furthermore, in those specific patients, high blood pressure resulted in a two-fold increase in relative risk of CV morbidity [39,49]. An escalation of 20 mm Hg of systolic blood pressure increased the risk for endothelial dysfunction and CVD.

Studies demonstrated that treatment in RA, disease-modifying anti-rheumatic drugs, DMARDs (especially leflunomide), non-steroidal anti-inflammatory drugs (NSAIDs), corticotherapy (CS), or cyclosporine, may be correlated with an increased risk for hypertension [50].

Other studies confirmed that there are no considerable discrepancies between RA and non-RA populations in terms of diagnosis and treatment of hypertension [51,52,53]. 

#### 3.1.3. Dyslipidemia 

Dyslipidemia is an important traditional risk factor for CVD in RA patients; it is believed that approximately 55–65% of the RA population presents a lipid metabolism impairment [54]. The state of active inflammation in RA is a key element implicated in the modulation of lipid metabolism, a traditional risk factor engaged in CVD appearance. There is a so-called “lipid paradox”, where the population having low levels of LDL-C has an increased CV risk comparing to those with higher levels [55,56]. Moreover, studies indicate that, in RA patients, total cholesterol and LDL-C values are decreased, in contrast with the non-RA population [57,58,59], despite RA being an independent risk factor for CVD [59].

Additionally, drugs used in RA treatment to reduce inflammation, such as DMARDs, TNFα inhibitors, and IL-6 receptor blockers (tocilizumab), may increase lipid levels [60]. In a study conducted by McInnes et al. [61], it was demonstrated that tocilizumab is connected to a reversal of IL-6–induced LDL-C clearance from the circulation, a fact also confirmed by Robertson et al. in another study from 2017 [59]. The Janus-kinase inhibitors’ (JAKi) impact on lipid changes is comparable to that of anti-TNFα and anti-IL-6 treatment. Tofacitinib is implicated in decreasing the LDL–C clearance from circulation and increasing serum cholesterol concentrations [62].

#### 3.1.4. Obesity

In RA, an association between low body mass and inflammation is described [60]. As previously shown in lipid metabolism, in RA, there is also an “obesity paradox”, with an inverse correlation between body mass index (BMI) and mortality. It is well-known that, in the general population, an increased BMI is associated with an important CVD risk; in contrast, in the RA population who are overweight or obese, there is a lower relative risk for mortality driven by a CV cause compared with patients with normal BMI [63]. A study by Escalante et al. demonstrated that the opposite association between BMI and mortality is no longer present after rectification for RA severity and comorbidities [64]. Thus, the “obesity paradox” may be explained by the effect of inflammation on involuntarily decreasing body weight. Considering that, Baker et al. conclude that weight is a better predictor than BMI for mortality in patients with RA [65].

#### 3.1.5. Insulin Resistance and Metabolic Syndrome

Studies indicate that the incidence of insulin resistance and metabolic syndrome is increased in patients with RA (40%), directly affecting endothelial dysfunction. A high amount of plasminogen activator inhibitor (PAI) and endothelin are found in plasma; thus, endothelial-dependent vasodilation is often compromised in people with insulin resistance [60]. 

There are four important adipokines that are recognized as being implicated in RA and CVD [66]: adiponectin, leptin, resistin, and visfatin.

**Adiponectin** is an adipokine known to have a pro-inflammatory role in RA patients [67]. High levels of adiponectin are associated with an increase in joint destruction [68]. 

**Leptin** is an inflammatory adipokine especially involved in controlling appetite and weight. It is also linked with inflammation in ATS, having an influence on some immune cells by inducing T-cell activation and endothelial dysfunction and stimulating fibroblasts to secrete inflammatory cytokines [69,70]. 

Serum **resistin** is normal in patients with RA, but its role on insulin resistance is given by high levels in synovial fluid; it is believed that this adipokine is implicated in increasing the permeability of the synovial membranes [69]. Resistin is involved both in joint inflammation and destruction and in endothelial impairment [67,71]. 

**Visfatin** is an adipokine with a demonstrated function of stimulating the secretion of inflammatory cytokines from monocytes and increasing IL-6, IL-8, and MMPs (Matrix Metalloproteinases) activity [67]. Studies show increased levels of visfatin in serum and synovial fluid in patients with RA [70,71], with negative consequences on joints and metabolism [66]. In addition, levels of this adipokine are important in CAD patients, being proportionate with disease severity [72]. 

#### 3.1.6. Reactive Oxygen Species 

Reactive oxygen species (ROS) are molecules implicated in endothelial disturbance in RA patients [50,73]. Levels of ROS in those individuals are increased, resulting in important cellular lesions; vascular endothelial cells are primarily affected, enhancing endothelial permeability and stimulating leukocyte adhesion. 

#### 3.1.7. Genetic Risk Factor 

It is well-known that RA and CVD have important genetic influences [74,75]. Genome-wide association studies (GWAS) have detected genes for RA and CVD, but there are not many common risk markers for those two [76]. 

A total of 55 disease-associated single nucleotide polymorphisms (SNPs) of RA and CAD are found by now [76]. In addition, Karczewski et al. [77] found some SNPs associated with RA and CAD within the nuclear factor kB (NFkB) binding sites. RA-specific genes (for example, TRAF1/C5, STAT4, and HLA-DRB1-shared epitope) are implicated in metabolism supervision in RA individuals [78]. At the same time, apolipoprotein E (APOE) genotypes are related to lipid levels in those patients, this way having a role in metabolism impairment [79]. Gene polymorphisms of IL-6–174 promoter and MTHFR (methylenetetrahydrofolate reductase) 1298 A1298C are related to high CVD risk in RA patients [80]. TNFα polymorphisms, rs180062965 and TNFα 1031 T/C, were discovered to be a potential risk factor for ATS in RA people [81]. At the same time, MHC (Major Histocompatibility Complex) molecules, such as the HLA-DRB1 gene, are thought to be involved in RA predisposition [82].

### 3.2. RA-Associated Risk Factors 

Chronic inflammation and pathological modifications present in RA are independent risk factors for ATS and may trigger the morbidity of CVD. Studies documented the relationship between the inflammatory burden and CVD risk in RA patients [37,83,84]. Inflammation pathways influence endothelial disturbance and atheroma plaque appearance. It is demonstrated that processes taking place in RA synovium and atherosclerotic plaques are similar [85].

During the inflammatory process, inflamed synovium and lymphoid tissues (represented by the spleen, lymph nodes, and adipose tissue) release proinflammatory cytokine into the blood flow, alongside complement activation [86]. In RA pathophysiology, these cytokines are major contributors, but they are equally implicated in endothelial dysfunction and other specific functions, such as insulin resistance, production of high levels of CRP, procoagulant factors, increased arterial stiffness, and atherosclerotic plaque development; eventually, all these aspects are implied in CVD in RA [87]. 

*TNFα* and its soluble receptors are implicated in the occurrence and progression of ischemia. They influence endothelial dysfunction and vascular instability, the incorporation of inflammatory cells at the injured location, and the process of reverse remodeling of vascular smooth muscle cells (SMC) [88].

The role of *IL-1* in vascular impairment is well-known. It induces the expression of adhesion molecules on endothelial cells and promotes neoangiogenesis. Additionally, IL-1 seems to be implicated in the function of different cells involved in heart injury and repair, thus facilitating apoptosis and hypertrophy of myocardial cells and inhibiting myocardium contractility [89].

*IL-6* is involved in different aspects related to vascular damage in RA: endothelial cell activation, increasing the formation of chemokines that attract T cells, and the appearance of VEGF (Vascular Endothelial Growth Factor). IL-6 enhances CRP production in the liver [90]. IL-6 also plays an important role in myocardial cells, with protective effects on cardiomyocytes during acute reactions. Even so, high levels of IL-6 may decrease the capacity of adaptability of myocardial cells, leading to hypertrophy and decreased systolic functions [91].

*IL-17* is a cytokine also studied and demonstrated to have strong implications in inflammatory pathways in RA and non-RA patients [7,92]. Increased levels of IL-17 are found in individuals with ACS. Microvascular function and arterial compliance are related to the activity of this cytokine in RA; for that reason, it may have a major function in the development of endothelial impairment and CVD [93].

*Complement activation*. The role of perivascular adipose tissue in generating complement proteins and the role of visceral adipose tissue in CAD are recognized [94]. In addition, complement activation products are able to influence the function of structural proteins from blood vessels and circulatory immune cells, further enhancing inflammation and the formation of atherosclerotic plaques [95]. Since the quantity of visceral adipose tissue is important in RA patients, it is believed that complement synthesis and activation might be implicated in CAD generation.

Studies have specified that RA patients with CAD have increased levels of terminal complement complexes, pentraxin 3 (mediator of the alternative pathway of complement activation), and important infiltration with mononuclear cells in the vessel wall compared with patients without RA [96].

**Figure 2:**
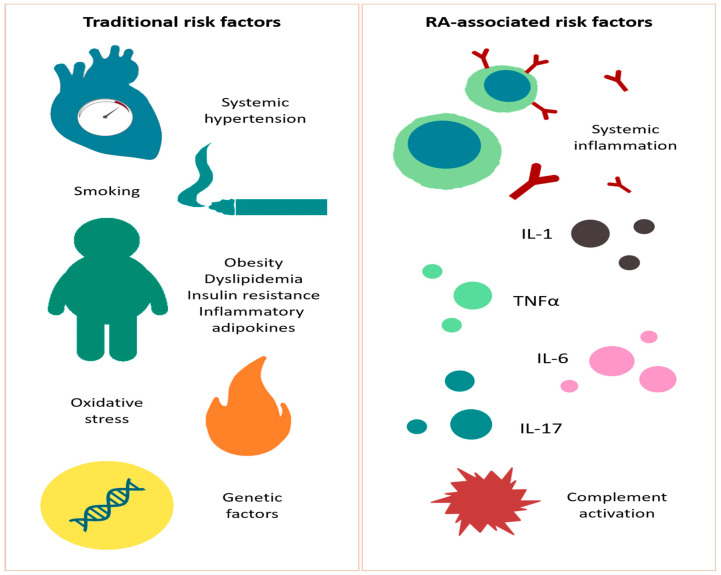
Cardiovascular risk factors. Smoking, hypertension, dyslipidemia, obesity, insulin resistance and metabolic syndrome, adipokines, reactive oxygen species, and genetic influences are the most prevalent traditional risk factors. Chronic inflammation and pathological modifications present in RA (rheumatoid arthritis) are independent risk factors for atherosclerosis and may trigger the morbidity of CVD; TNF (tumor necrosis factor) α, IL (interleukin)-1, IL-6, IL-17, and complement activation represent RA-associated risk factors.

Figure 2 schematically explains the most important cardiovascular risk factors in RA.

## 4. Inflammation and Atherosclerotic Burden in Rheumatoid Arthritis

### 4.1. Accelerated ATS in RA

As previously shown, the immune system is implicated through various mechanisms in atherogenesis, and the presence of immune cells in atherosclerotic plaques proves this concept [97,98]. A lot of constituents of the immune system are part of the atherogenesis process, TNFα and IL-1β being the most essential ones [99].

### 4.2. Endothelial Dysfunction

The vascular endothelium is involved in the regulation of physiological processes taking place in vessels, such as vascular tone, inflammatory response, and coagulation. Damage of the endothelium is the key event in atherogenesis [100], leading to an increase in permeability to inflammatory cells. Furthermore, increased expression of adhesion molecules and cytokines are correlated with endothelial dysfunction, encouraging the infiltration of monocytes in the sub-endothelial level [101].

The balance between vasoconstrictors (angiotensin II (ANG-II), thromboxane A2 (TxA2), and endothelin-1) and vasodilators (prostacyclin (PGI2), nitric oxide (NO) and endothelium-derived hyperpolarizing factors (EDHFs)) is directly responsible for the proper function of the endothelium. Studies indicate that inflammation is accompanied by high levels of COX (cyclooxygenases)-2, and it is well-known that TxA2 and PGI2 are vascular regulators produced by COX [102].

### 4.3. Plaque Development

**Figure 3:**
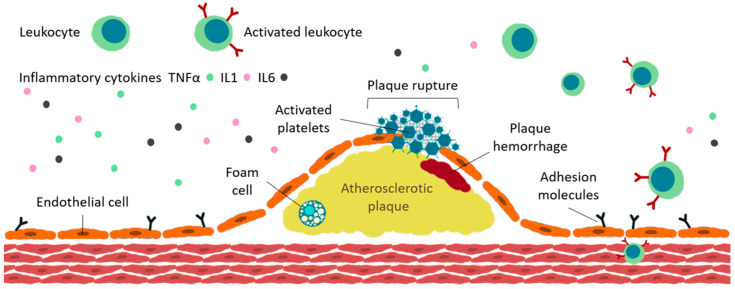
Atherosclerosis (ATS) plaque formation. Immune system is implicated through various mechanisms in atherogenesis; the presence of immune cells (such as TNFα, IL-1, and IL-6) in atherosclerotic plaques proves this concept. Vascular endothelium is involved in regulation of physiological processes taking place in the vessel. The first step in the development of atherosclerosis is the accumulation of low-density lipoproteins (LDLs) and leukocytes into the sub-endothelium. LDLs are modified and become aggregated and/or oxidized. The monocytes present in the sub-endothelium layer differentiate to macrophages, which will engulf Ox-LDL, thus determine the accumulation of cholesterol in macrophages and formation of foam cells, which will lead to increased secretion of cytokine and chemokine and further recruitment of circulating immune cells. High levels of endothelial adhesion molecules, such as vascular cell adhesion molecule-1 (VCAM-1), monocyte chemoattractant protein-1 (MCP-1), and intracellular adhesion molecule-1 (ICAM-1) are identified. Apoptosis and necrosis are stimulated by high levels of cholesterol in foam cells and, after cellular death, cholesterol esters will accumulate and produce atherosclerotic plaque. The most frequent cause of thrombosis is plaque instability and then fracture. Once the fibrous cap is ruptured, the substances from the plaque are released and put in contact with blood; this may cause obstruction of blood flow and, in the end, the specific symptoms of thrombosis. TNF, tumor necrosis factor; IL, interleukin (after [130]).

Figure 3 explains the most important aspects related to ATS plaque formation.

The first step in the development of ATS is the accumulation of LDLs and leukocytes (mostly monocytes) into the sub-endothelium [103]. Alteration of endothelial permeability and paracellular transport between modified cells allows them to move in the sub-intimal space; recent studies discovered another mechanism, represented by active receptor-mediated transcytosis across the cell membrane, using specific transporters [104].

Following this process, LDLs are modified and become aggregated and/or oxidized. Thus, large complexes ranging in size from 100 nm to 1.0 mm appear and will suffer phagocytosis or pinocytosis by immune cells already existing there [105]. Additionally, LDLs are oxidated, converted into Ox-LDL. The monocytes present in the sub-endothelium layer differentiate to macrophages, which will engulf Ox-LDL, thus determining the accumulation of cholesterol in macrophages and formation of foam cells [106]. Foam cells will lead to increased secretion of cytokine and chemokine and further recruitment of circulating immune cells [106]. Following this, high levels of endothelial adhesion molecules, such as vascular cell adhesion molecule-1 (VCAM-1), monocyte chemoattractant protein-1 (MCP-1), and intracellular adhesion molecule-1 (ICAM-1) are identified; in the end, all these changes will stimulate further leukocyte recruitment and infiltration to the endothelium [107]. 

Apoptosis and necrosis are stimulated by high levels of cholesterol in foam cells and, after cellular death, cholesterol esters will accumulate and produce atherosclerotic plaque [108,109]. TNFα stimulates the secretion of chemokines and the formation of scavenger receptors (SRs) on macrophages, thus increasing the expression of acyl-coenzyme A: cholesterol acyltransferase (ACAT) and further foam cell development [110]. 

The presence of specific antibodies to Ox-LDL (anti-oxLDL) in the sera of patients with rheumatic diseases is also important, and they are higher than in the general population. These antibodies are good predictors of peripheral vascular involvement and the severity of ATS [111]. It is also important to mention other autoantibodies correlated with ATS [112], for example, antibodies against heat shock proteins (HSPs) from the surface of endothelial cells exposed to stress or antinuclear antibodies (ANA) found especially in patients with symptomatic angina pectoris [113].

Another important aspect of ATS plaque formation is the implication of SMC from the media layer of the artery. Due to the influence of transformed cholesterol, growth factors, and cytokines implicated in the process described above, SMC will change from a primarily contractile, non-proliferative genotype to a proliferating one, with migratory and matrix secreting properties in the arterial intima. Recent searches confirm the role of SMC in inflammatory events in the ATS plaque, suggesting that up to 50% of the cells present in such a structure result from an SMC lineage [114]. 

### 4.4. Plaque Destabilization 

The most frequent cause of thrombosis is plaque instability and then fracture. Once the fibrous cap is ruptured, the substances from the plaque are released and put in contact with blood; this may cause obstruction of blood flow and, in the end, thrombosis-specific symptoms [115]. 

### 4.5. Lipid Metabolism

After the accumulation of LDL-C in the vascular sub-endothelium and the increased absorption by macrophage cells, the status of LDL-C in serum is significantly increased, with concomitant high levels of cholesterol. It is known that HDL-C outflow is a process that prevents the storage of cholesterol in macrophages. In rheumatoid diseases, HDL-C and cholesterol homeostases are impaired [116,117]; this mechanism will contribute to atherosclerotic macrophages formation. This is another confirmation of atherogenic factors’ presence in RA patients and the participation of the immune system in ATS [118,119].

### 4.6. Micro- and Macrovascular Involvement in RA

Both microvascular and macrovascular alterations are identified in RA patients and are linked to enhanced CV risk in those individuals [120], though studies did not confirm an important interrelation between the two processes.

#### 4.6.1. Macrovascular Involvement in RA 

cIMT (carotid intima-media thickness) is raised in patients with RA, being related to disease duration and the age of the patients. However, studies did not confirm a sustained connection between inflammation and vascular parameters. Carotid plaque is an important feature studied in RA patients. Roman et al. reported that carotid plaque was more frequent in RA patients than in controls. Additionally, in another study conducted by Dessein et al., in 31% of such individuals, carotid atherosclerotic plaques were present [121], compared to 35% found in a study realized by Pope et al. [122] RA CV risk evaluation is very important, and carotid and femoral ultrasonography is the main investigation.

As discussed earlier, plaque rupture is the central event leading to clinical symptoms. The structure of the plaque (especially the presence of calcification, a lipid-rich necrotic core, neovascularization, or the existence of inflammatory cells) and inflammation are the principal risk determinants for such incidents [123]. In a study by Skeoch et al., it is demonstrated that the atherosclerotic plaques in RA patients have an increased chance of developing complications [124]. 

#### 4.6.2. Microvascular Involvement in RA 

The relationship between inflammation, immune malfunction, and traditional CV risk factors is the trigger for microvascular endothelial disorder in RA patients [125]. Galarraga et al. indicated that systemic inflammation (assessed by CRP levels) is an independent risk factor for microvascular dysfunction in RA patients [126]. In another study, Arosio et al. illustrated that patients with RA outlined compromised microcirculatory reactivity, endothelial dysfunction, and increased arterial stiffness, such elements being directly connected with increased levels of CRP and inducible NO synthase [127]. 

Nailfold capillaroscopy is a non-invasive and replicable method for the morphological evaluation of microvascular aspects. Thus, we can assess tortuosity, loop size, density, capillary loss, angiogenesis, microbleeding, subpapillary venous plexus, and architectural configuration. Altomonte et al. described that elongation and capillary tortuosity are the main capillaroscopic changes in RA patients. Additionally, the visibility of the subpapillary venous plexus was interrelated with endothelial dysfunction. In another research, Lin et al. outlined that, in the RA population, elongated and tiny capillaries and capillary tortuosity are the most specific discoveries and that the subpapillary venous plexus was present mostly in RA patients who were ANA-positive [128]. Cutolo et al. indicated that a “scleroderma-like” capillaroscopic pattern might be described in patients with RA, particularly when rheumatoid vasculitis is present [129]. 

## 5. Impact of Medication on Development of Ischemic Heart Disease in Rheumatoid Arthritis

In RA, treatment objectives are to diminish pain and inflammation, prevent joint destruction, and reduce disability. The main goal is, in fact, to achieve full remission, or at least a low disease activity. 

Current therapies for RA are represented by NSAIDs and CS (efficacious for managing pain, inflammation, and stiffness), conventional synthetic DMARDs (csDMARDs), as first-line therapy, and targeted synthetic DMARDs (tsDMARDs) and biological DMARDs (bDMARDs) that inhibit specific molecules involved in the immunological cascade in RA. 

### 5.1. NSAIDs and CS

Long-term use of *NSAIDs* is confirmed to increase CV risk. They are involved in COX isoforms inhibition [131,132]. The consequence of COX-2-mediated prostacyclin suppression is vasoconstriction, platelet mobilization, atherogenesis, and increased risk for hypertension, which is deemed as a principal risk factor for CVD in RA [133]. In reality, there is a disproportion between PGI2, which functions as a vasodilator and inhibitor of platelet aggregation, and TxA2, known as a vasoconstrictor and initiator of platelet aggregation because of the selective cyclooxygenase blockage that raises the risk of CVD [134].

Moreover, atherosclerotic plaques present high levels of COX-2 due to the macrophages, vascular SMCs, and endothelial cells activity, so it is postulated that increased COX-2 is a part of the atherosclerotic inflammatory mechanism. Therefore, COX-2 suppression may have positive results in decreasing the risk for ATS [135].

In 26 trials studied, NSAIDs were responsible for 46% of deaths caused by CV events. A meta-analysis performed by Telle et al. investigated CV risk in seven NSAIDs. Naproxen had the lowest risk, as the other six (ibuprofen, diclofenac, lumiracoxib, celecoxib, etoricoxib, and rofecoxib) revealed the same risk profile in terms of CVD. The same study indicated that the risk of death connected to NSAID use is higher at the initiation of the treatment [25]. In another paper, Kimmel et al. showed that among the NSAIDs, diclofenac and indomethacin had the most prominent risk for CV events, with ibuprofen interfering with the cardioprotective effects of aspirin [136]. For RA patients, EULAR guidelines specify that, for CAD risk, naproxen is the safest [15]. 

Regardless, when prescribing an NSAID to a patient with CV and gastrointestinal risk factors, we also have to consider the variety of NSAIDs, the imbalance in COX-1/2, and the level of inhibition, as well as the proper dose and duration of treatment in those individuals. Further, it is important to note that aged patients have a superior risk for CVD, so, in that case, NSAIDs should be prescribed at the lowest efficient dose and for the shortest time possible [137]. 

*CSs* in RA patients are indicated for a short period of time in order to control disease activity [137]. They are able to induce or aggravate the well-known traditional CV risk factors [138], causing or worsening hypertension, influencing blood lipid levels and insulin resistance, and increasing abdominal obesity, thus facilitating the onset and the development of CVD [137]. For this reason, it is imperative to prescribe CSs in small doses and for short periods of time, as the present guidelines advise.

Studies have proved that in RA patients, CSs enhance the risk for CV events, including MI, HF, cerebral infarction, and major adverse cardiac events (MACE) [139]. Nurmohamed et al., in a meta-analysis from 2015 assessing CS treatment in RA, stated that prednisone or prednisone-equivalents taken for long periods of time and in high doses (7.5 mg per day or more) are related to a higher risk for CV mortality [140]. 

Therefore, EULAR recommends administrating the lowest effective doses of CS, to taper them when is possible (in case of remission or low disease activity, the main goals of therapy), and be aware of any adverse effects during treatment [141,142]. 

### 5.2. csDMARDs

csDMARDs are the first-line- pharmacological treatment in RA and are represented by methotrexate (MTX), sulphasalazine, leflunomide, gold salts, and hydroxychloroquine [143].

*MTX* is the “gold standard” for RA treatment. It plays an important immunosuppressive and anti-inflammatory effect, inhibiting dihydrofolate-reductase. The beneficial effects of MTX are confirmed in many studies, as approximately 25–40% of patients improve considerably with MTX in monotherapy; in combination with CS, the percent of remission is comparable to that of bDMARDs. Additionally, MTX increases bDMARDs’ therapeutic success [143,144].

CV consequences of this medication have been investigated for a long time. The CV risk for MTX is minimal; in fact, MTX may decrease CV events by 21%, as shown by a meta-analysis realized by Micha et al. [145]. Research published by Choi et al. indicated that RA patients treated with MTX had a 60% reduction in all-causes deaths and a 70% reduction in CV deaths [146]. Vascular impact of MTX is also important but is still not very well established. On the one hand, it stimulates the formation of pro-atherogenic homocysteine, which is toxic for endothelial cells and enhances LDL-oxidation, but on the other hand, it suppresses inflammatory pathways, with positive CV outcomes [15,138,147]. 

MTX exhibits convenient effects on several markers of CVD, increasing reverse cholesterol transport, lowering foam cell [148] and adhesion molecule formation, and decreasing the risk for metabolic syndrome [66]. Besides, pro-inflammatory and pro-atherogenic cytokines, such as TNFα, IL-6, and IL-1, are impaired. Studies have also demonstrated satisfying effects on ATS progression [149]. 

*Antimalarial drugs* (especially hydroxychloroquine, HCQ) have a poor CV risk in RA patients; actually, it is believed to have cardioprotective effects [138,150,151]. HCQ treatment has a good impact on lipid profile (with reduced LDL and triglyceride serum values) and exerts antithrombotic effects on platelet aggregation, with minimal risk for diabetes mellitus [152]. Furthermore, HCQ plays important functions related to cytokines production and T cells and monocytes activity. A meta-analysis provided by Mathieu et al. showed that patients with RA treated with HCQ have fewer CV issues than non-HCQ RA individuals [153]. 

Studies suggest that *sulfasalazine* can diminish CV risk in patients with RA [154,155]. In a case–control study communicated by van Halm et al., treatment with sulfasalazine exhibited a lower risk for CV problems than RA patients without any csDMARD therapy [155]. 

Leflunomide is the cause of high blood pressure, exacerbating hypertension. Additionally, it is demonstrated to decrease the risk for MI comparing to RA patients taking other csDMARDs [151]. 

### 5.3. bDMARDs 

bDMARDs are blocking pro-inflammatory cytokines (TNFα, IL-6, or IL-1) or pathogenic immune cells, which are involved in inflammatory diseases and atherogenesis [156]. The essential role of these bDMARDs in inhibiting the process of inflammation in active RA (and in other rheumatological diseases) caused clinicians to study the relationship between this class of medication and CVD more deeply.

#### 5.3.1. Anti-TNFα 

TNFα is a cytokine that plays a central role in the inflammatory cascade in RA, controlling the immune response and influencing cellular and humoral immunity [157]. The five TNF inhibitors (TNFi) used in RA are: infliximab (a chimeric anti-TNFα antibody), adalimumab and golimumab (fully humanized anti-TNFα antibodies), etanercept (a fusion protein linked to the Fc region of human antibody), and certolizumab pegol (a modified human antibody) [5]. 

Anti-TNF treatment has been established to lower the CV risk in RA patients, influencing the atherogenesis process and endothelial cells activation, thus ameliorating cardiac function. However, this reduction of risk for CV events does not always include HF [139]. FDA made a recommendation against the use of TNFi in RA patients with CHF (congestive heart failure) [158]. A meta-analysis revealed that treatment with TNFi has favorable effects on CVD, notably increasing the risk for CV events, MI, stroke, or MACE; also, the risk for CAD may be lowered after long-term treatment with TNFi [15,159].

Blocking TNFα activity has good results on traditional risk factors for ATS, for example, metabolic syndrome and insulin resistance [160]. Daien et al. revealed that anti-TNF treatment especially affects the level of HDL-C, recovering its anti-atherogenic capacity [161]. Other researches specify that this therapy increases total cholesterol and triglycerides (and HDL-C, as previously revealed) without any consequence on LDL-C or on the atherogenic index after long-term treatment, denoting a potential protective effect on lipid metabolism [161,162].

Ljung et al. published a study demonstrating that, regarding RA biological treatment, good responders to TNFi therapy have a superior risk for ACS than moderate or non-responders, but the risk for such events in patients with a positive response to therapy is not substantially different from that of the general population; also, the risk of moderate responders or non-responders can be up to more than twice as high [163].

It is important to mention that the risk for hypertension is another serious side effect of TNFα antagonists’ treatment in RA patients [164]. 

Several investigations proved that TNFi in RA individuals has satisfying results in ameliorating arterial stiffness (expressed as dropping of pulse wave velocity, PWV) and endothelial dysfunction (analyzed as improvement in flow-mediated vasodilation, FMD) [165,166]. In rheumatic diseases, anti-TNFα therapy inhibits cIMT progression [166], but the results are more significant in RA patients with early disease onset [167].

Subclinical ATS is an important issue that may concern CV risk in RA patients. Different surveys illustrate that TNFi has separate effects; adalimumab and etanercept perform an important diminution of arterial stiffness, albeit there is no modification with infliximab treatment [168].

#### 5.3.2. Anti-IL-6 

IL-6 is a proinflammatory cytokine derived from T lymphocytes, macrophages, and adipocytes, which stimulates the synthesis of hepatic acute phase reactant production (CRP and fibrinogen) and facilitates ATS. Additionally, IL-6R is implicated in the progression of CAD [169]. 

Tocilizumab (TCZ) is a monoclonal antibody, inhibitor of the IL-6 receptor, whose efficacy and safety in RA has been previously proven, but TCZ is also able to influence lipid metabolism by increasing LDL-C and total cholesterol [170] and decreasing HDL-C [168]. Studies, for example, MEASURE and ADACTA, support these results. The risk for MACE during TCZ treatment in patients with RA is not linked to lipid homeostasis alteration but to disease activity [171,172]. 

In a recent meta-analysis, Singh et al. revealed that TCZ was not found to be associated with an increased CV risk compared to TNFi [173]; the same results were also illustrated by the ENTRACTE trial, comparing TCZ to etanercept in patients with active RA and CV risk factors [174]. Kobayashi et al. showed that TCZ might provide a protective role in SCD, knowing that RA individuals have a two-fold higher risk for this complication as a result of QTc interval prolongation [175]. 

The TOCRIVAR clinical trial reinforced the idea that TCZ in RA patients performs a convenient effect on lipid metabolism and CV risk. The conclusions of this study are relevant in this direction: long-term TCZ treatment increasing the CEC (cholesterol efflux capacity) of macrophages, proportional with CRP reduction; further, TCZ is responsible for lowering Lp(a) levels, an independent risk factor for CVD [176]. 

Biological treatment with TCZ has been correlated with amelioration of endothelial function (measured with FMD) and arterial stiffness [167], but no significant changes were mentioned in cIMT [177]. 

Kim et al. developed a multidatabase cohort study, suggesting that CV risk is equivalent to TCZ treatment compared to TNFi in RA patients [178].

#### 5.3.3. Anti-CD-20 

Rituximab (RTX) acts as a humanized chimeric anti-CD20 monoclonal antibody, playing a determinant role in blocking B cell activation. Studies showed a similar CV safety profile to TNFi [5,179]. They even considered a positive effect of RTX on CV risk, inhibiting antibodies implicated in atherogenesis, interfering with vasoconstriction process, thrombocytes activation, and atherosclerotic plaques’ rupture [156].

In relation to the vascular involvement of RTX, clinical trials demonstrate an improvement of cIMT but without a notable effect on arterial stiffness [180]. Moreover, even if there was a minor increase in total cholesterol and triglyceride serum levels, RTX treatment ameliorated both macrovascular and microvascular function of the endothelium in relation to brachial artery flow-mediated dilation and reactive hyperemia velocity [181].

The risk of MACE was similar between RTX and TCZ in a study performed by Gottenberg et al. [182], but more information is needed when describing the definite effect of RTX on CAD.

#### 5.3.4. Anti-IL-1

IL-1 is another pivotal cytokine implicated in the inflammatory cascade from RA and in ATS development, alongside TNFα and IL-6. IL-1β is the circulating form of IL-1. Canakinumab, an IL-1β monoclonal antibody, and anakinra, an IL-1 receptor antagonist, are the two IL-1 inhibitors studied in relation to CV risk.

Data concerning the relation between IL-1 antagonists and the risk for CV events were obtained from CANTOS (Canakinumab Anti-inflammatory Thrombosis Outcomes Study). A total of 10,061 men and women with a history of MI and high hs-CRP (high sensitive-CRP) levels were randomized to receive three doses (50 mg, 150 mg, or 300 mg) of canakinumab or placebo given subcutaneously once every 3 months. Patients with canakinumab had considerably lower values of serum hsCRP compared to placebo; in addition, individuals taking 150 mg/3 months exhibited an increased number of CV events, but without any change in lipid levels or platelet function [183,184]. 

Another important study, conducted by Ikonomidis et al., suggested that anakinra is capable of reducing oxidative stress in patients with RA, thereby augmenting the physiology and structure of the cardiac and vascular system. RA patients with CAD treated with anakinra experienced an increased left ventricular ejection fraction [185].

#### 5.3.5. Anti-CD80/86

Abatacept blocks the activation of T cells by selectively inhibiting the specific binding of CD80/CD86 to the CD28 receptor, thereby influencing T cell proliferation and immune responses of B lymphocytes. Studies indicate that abatacept exhibits a similar risk of MACE compared to TNF inhibitors [173]. In a study published by Generali et al. in 2019, abatacept does not influence the risk for HF when compared with etanercept [186].

### 5.4. tsDMARDs—JAK Inhibitors

JAKi are a class of drugs that represents a new category of treatment known as tsDMARDs. The JAK family of enzymes is connected with cytokine receptors existing on the surface of cells. They are part of the Signal Transducer and Activation of Transcription (STAT) pathway, implicated in immune responses and inflammation [187]. 

There are insufficient data concerning the impact of the two JAKi (baricitinib and tofacitinib) on CV risk in RA. It is considered that both aggravate lipid profiles, as shown in two studies, one by Kremer et al. and another by Charles-Shoeman et al., demonstrate that treatment with JAKi produces increased levels of LDL-C, HDL-C, and triglycerides [62,188]. Even so, other clinical studies do not indicate a negative impact on CV risk in patients with RA and JAKi treatment [189]. 

Researchers showed that, in RA patients treated with tofacitinib, HDL-C levels were higher, and the total cholesterol to HDL-C ratio was lower. This situation is correlated with a reduced risk for eventual MACE. Additionally, increased levels of total cholesterol and LDL-C failed to have these protective results [190]. 

A recent study by Taylor et al. showed no direct correlation between baricitinib treatment and the occurrence of arterial thrombotic events, CHF, or MACE. During the study, 6 patients treated with 4 mg baricitinib, with previous risk factors for venous thromboembolism, experienced deep vein thrombosis or pulmonary embolism [189]. However, there is still major interest and worry regarding venous thromboembolisms following JAKi therapy.

## 6. Assessing Cardiovascular Disease Risk in Rheumatoid Arthritis

### 6.1. CVD Risk Scores

In the general population, there are available CVD risk scores, such as Systematic Coronary Risk Evaluation (SCORE), Framingham Risk Score (FRS), Reynolds Risk Score (RRS), and the Expanded Cardiovascular Risk Prediction Score for RA (ERS-RA), all evaluating traditional risk factors (age, gender, lipid levels, smoking, hypertension, impaired glucose tolerance) [191,192,193]. 

The **SCORE** system represents a CV risk evaluation instrument created for the general population that includes both non-changeable (age, gender) and changeable items (lipid status, smoking, and blood pressure). It was elaborated in 1994 by the ESC and the European Society of Hypertension (ESH) and included a population 40 years old or more. Based on the results obtained, patients were distributed into four risk groups, <1%, 1–4%, 5–9%, and ≥10%, and in each one, incorporated primary (LDL) and secondary (non-HDL-C and Lp(a)) therapeutic targets were incorporated. [194]. This method underrated the risk for developing a CV event in RA patients [191,195]. It is important to mention that there are a lot of disadvantages when realizing SCORE evaluation in patients with RA. Even when the disease is inactive, lipid profile (mainly total cholesterol and triglycerides) must be analyzed [196,197]; in the active period of RA, the ratio of total cholesterol to HDL-C is more predictable [198]. 

EULAR agreed with multiplication by 1.5 to the SCORE system in RA patients in clinical practice. This adjustment is utilized for all RA patients, no matter of disease-related criteria. A study by Crowson et al. indicated that the 1.5 multiplication proposed by EULAR is not a better or a more appropriate predictor of the risk for CV episodes when considering the RA population [199]. EULAR recommends an evaluation of risk every 5 years, in case of a low or moderate initial calculated risk, or more frequently (once a year), if there is an intermediate or high risk [200]. Changes in lifestyle and specific therapy with lipid-lowering medication are required when CVD risk transcends a 10-year risk of 5% for fatal CVD events calculated with SCORE.

The **RRS** represents an instrument that considers the traditional cardiovascular risk factors and the patient family history of myocardial ischemia and hs-CRP [200]. Age, total cholesterol levels, HDL-C, systolic blood pressure, smoking, MI in family members under 60 years of age, hs-CRP, and glycosylated hemoglobin levels were part of the factors studied [201,202].

The **FRS** is a score made public in 1998 and has two versions [203]. The first version estimates the 30-year risk for CV events and contains, as factors evaluated, gender, age, systolic blood pressure, antihypertensive treatment, smoking status, type 2 diabetes, total cholesterol level, and level of HDL-C or BMI. The second version integrates age, diabetes, smoking, antihypertensive treatment, level of total cholesterol, level of HDL-C or BMI, and calculates the 10-year risk for CVD [191]. Patients are rated in three risk groups: <10%, between 10% and 20%, and over 20%.

Studies show that in RA patients, FRS can also underestimate the CVD risk. This trial demonstrated that documented CV risk in such population was higher than the predicted one, especially in seropositive patients with high levels of ESR and more than 75 years old [204]. Both FRS and RRS failed to determine the risk for cardiac and vascular issues in RA women with increased levels of CRP. It is believed that the FRS diminished the risk by 65% in men and 102% in women.

In order to estimate CVD risk in patients with RA, the investigators created **ERS-RA**, based on the CORRONA registry, which also comprised RA disease activity and duration, level of physical disability, and prednisone use. However, ERS-RA achieved similar results as FRS and RRS, so this score was not validated [205,206]. 

Other elements should be considered, for example, diabetes mellitus and chronic renal disease or some individual treatments. Family medical records, race, and psychological and sociocultural factors are also important. BMI is the instrument used to quantify body weight; however, in patients with RA taking CS, it may not be the wisest decision, so, in such individuals, we can measure the body fat from the abdomen (intra-abdominal fat). An additional issue in RA is the potential cachexia induced by long-lasting disease activity [207,208].

### 6.2. Imaging Modalities 

Imaging methods are indispensable for CV risk evaluation in RA, patients with this disease being known to have unstable coronary atherosclerotic plaques, an increased cIMT, and an affected myocardial function. Subclinical CV involvement in patients with RA may be assessed using CT (for coronary artery calcification) [208], carotid ultrasound (for IMT) [209], and aortic pulse wave velocity or arterial augmentation index and ankle–brachial index (for arterial stiffness)[210], echocardiography and cardiac MRI [211]. 

Evaluation of arterial changes (which appear early in CVD) in patients with RA is of clinical importance, highlighting further CV risk for those individuals. The ultrasound method enables a non-invasive achievement of images that show specific aspects of the vascular system and changes that may appear early in RA development. This detailed evaluation of the vessels correlates with and is influenced by CV factors (traditional or non-traditional), so ultrasound examination may provide useful information regarding CVD risk stratification, contributing to optimal treatment [212,213,214,215]. 

Carotid ultrasound is the most used and analyzed imaging tool in assessing CVD in RA. Studies illustrated that presence of carotid plaques in RA is related to disease duration and activity, but also with IHD [122,216]; therefore, this is the only method introduced in EULAR guidelines for clinical practice.

In two clinical trials published by Evans et al. and Corrales et al., the importance of arterial ultrasound in CVD evaluation in RA individuals is demonstrated. In the first study, 599 patients with RA and no history of ACS were randomized; the bilateral carotid plaques showed an increased risk for ACS (four times higher), and the relation between RA, and this syndrome was irrespective of traditional or disease-related risk factors [217]. Moreover, the occurrence of bilateral carotid atherosclerotic plaques was correlated with an enhanced risk for CV incidents [218]. 

cIMT ultrasound evaluation is easy to perform, affordable, viable, applicable, and replicable, and, most important, it correlates with atherosclerotic changes in RA [219,220]. It is a safe method used equally in the general population and in clinical studies in order to investigate the evolution of the ATS process. It is reported that many CV risk factors are positively connected to cIMT, such as age, BMI, blood pressure, elements of lipid profile (triglyceride and LDL), inflammatory syndrome (evaluated by ESR and CRP), hemoleucogram (especially hemoglobin, hematocrit, and platelet levels), creatinine, and uric acid. Further, there is an opposite rapport between cIMT and HDL-C [219].

Arterial stiffness appreciation with the help of ultrasound is likewise important in CVD risk stratification. The results obtained by measuring aortic pulse wave velocity and augmentation index may be predictive for CVD in the general population and in RA patients; disease activity can influence these results [211]. As shown in clinical trials, peripheral arterial stiffness (determined using the brachial–ankle elasticity index) is altered in individuals with RA disease and is a prognostic item for CVD morbidity and mortality in the general population [221]. 

In addition, there are two other trusted measures of coronary artery atherosclerosis, CT coronary angiography and coronary artery calcium scores, which were approved for CVD appraisal [222,223]. Aspects found in CT evaluation of ATS are related to RA disease activity, but using them as prognostic instruments is restricted [224].

Besides the detection and evaluation of ATS, echocardiography and cardiac MRI are essential in describing subclinical structural and functional deficiencies responsible for CHF [225,226,227]. 

**Figure 4:**
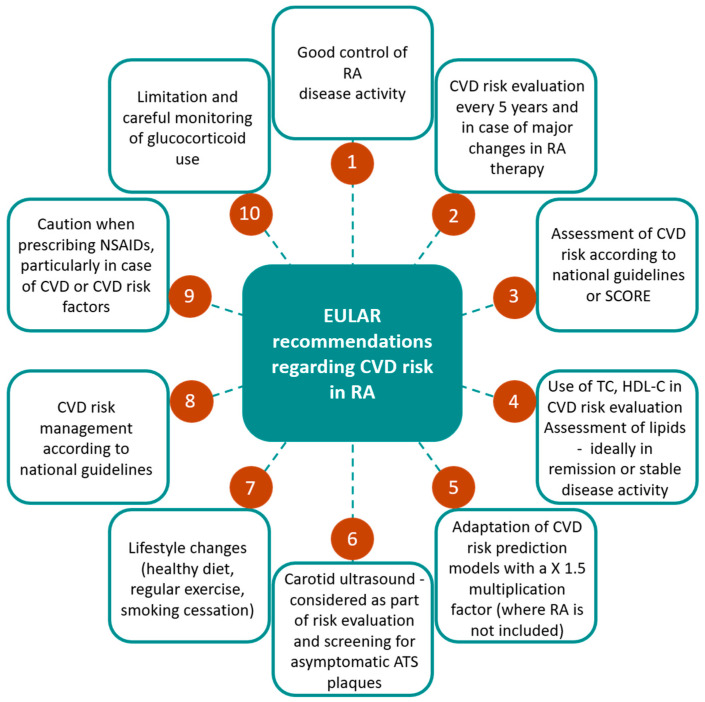
EULAR recommendations for cardiovascular risk evaluation in RA. EULAR, European League Against Rheumatism; ATS, atherosclerosis; CVD, cardiovascular disease; HDL-C, high-density lipoprotein—cholesterol; NSAID, non-steroidal anti-inflammatory drug; RA, rheumatoid arthritis; SCORE, systematic coronary risk evaluation; TC, total cholesterol.

EULAR recommendations for CV risk evaluation in RA are presented in Figure 4 [15]:

## 7. Conclusions

Observational data confirm that patients with RA have an increased risk for CVD and CV-related morbidity and mortality compared to the general population. Additionally, RA is described as an independent risk factor for CAD because of the inflammation that underlies the disease. It is considered that CVD is present in approximately 30% of RA patients 40 years and older, representing the most frequent cause of early death. Cardiac and vascular involvement in RA is multifactorial. Traditional CV risk determinants and RA-specific features are implicated in the excess of CV events in RA.

The involvement of chronic systemic inflammation in the pathophysiology of ATS is elaborate, and multiple mechanisms have been described. The accelerated atherogenesis process is probably the main cause for the elevated risk of ischemic issues in RA patients; thereby, it is mandatory to evaluate and determine any signs of subclinical ATS using non-invasive measures, such as cIMT or inflammatory markers.

In RA, an approved algorithm for CVD risk evaluation is missing. Existing scores for CVD assessment in the general population do not include factors linked to RA. Currently, different scores have emerged, incorporating RA- specific factors, but studies to confirm their validity are lacking. SCORE is one of the instruments that quantifies CV risk in everyday clinical practice in the general population, though in RA, it has some limitations. For patients with RA, EULAR proposed to multiply the results obtained by 1.5. Until RA-specific CVD risk assessment tools are identified, it is advised to use the national CVD risk scores, with an interval of evaluation named for each of the CV factors examined.

It is a constant challenge for physicians to correctly evaluate the mechanisms of CV risk in every RA patient. 

Treatment of RA has developed in recent years, with many patients being in remission or having low disease activity, as a result of the treat-to-target approach, which efficiently also decreases CV risk. Present EULAR guidelines emphasize the role of rheumatologists in measuring and standardization of CVD risk management in patients with RA, and accentuate the importance of suppressing chronic inflammation. In addition, EULAR outlines the idea of closely monitoring the traditional risk factors for CVD in those patients in order to minimize possible complications of the CV system. Nowadays, it is suggested that, in order to reduce the possibility of CV events in RA and to ameliorate disease activity, treatment should aim for proper control of inflammatory pathways and correct outlook for traditional CV risk factors.

Despite the earlier diagnosis and improved and adapted RA management, there are still subjects without or with poor response to therapy. They fail to reach treatment goals, and their CV involvement remains an interest for clinicians, as it also concerns patients’ morbidity and mortality. 

## Data Availability

Not applicable.
